# Perspectives on Implementation: Challenges and Successes of a Program Designed to Support Expectant and Parenting Community College Students in Rural, Midwestern State

**DOI:** 10.1007/s10995-020-02879-6

**Published:** 2020-02-06

**Authors:** Natoshia Askelson, Grace Ryan, Felicia Pieper, Whitney Bash-Brooks, Addie Rasmusson, Mary Greene, Amy Buckert

**Affiliations:** 1 Public Policy Center, University of Iowa, 310 S. Grand Ave, Iowa City, IA 52242 USA; 2 College of Public Health University of Iowa, 145 N. Riverside Dr., Iowa City, IA 52246 USA; 3 Bureau of Family Health, Iowa Department of Public Health, 321 E 12th St, 6th Floor, Des Moines, IA 50319 USA

**Keywords:** Community college, Parenting, Implementation evaluation, Student parents

## Abstract

**Objectives:**

Expectant and parenting students (EPS) at community colleges are an underserved and often under-resourced group. In a rural, Midwestern state, the department of public health was awarded the Pregnancy Assistance Fund (PAF) grant to assist this population. This paper outlines the results of the implementation evaluation and offers suggestions for programs and evaluators working with this population in the community college setting.

**Methods:**

We conducted a multicomponent evaluation utilizing quantitative and qualitative methods. Evaluation activities included tracking activities/services, surveys and interviews with participants, and interviews with community college staff implementing grant activities. The research team calculated frequencies for quantitative data and coded qualitative data for themes.

**Results:**

Data from the community colleges and students’ self-reports revealed that EPS most commonly received concrete support from the program, often in the form of stipends or gift cards. Students reported that concrete support was beneficial and helped to relieve financial stress during the semester. Students’ major barriers to participation were lack of knowledge about the program and busy schedules that prevented them from accessing PAF services. Staff reported that difficulty identifying EPS and the short one-year project period were major implementation challenges.

**Conclusions for Practice:**

We recommend that community colleges work to identify EPS, use fellow EPS to recruit program participants, and implement programming that works with the students’ schedules.

## Significance

Very little is known about the needs of EPS at community colleges and how to develop beneficial programming for them. EPS commonly attend community colleges and all EPS face barriers to graduation that their non-parenting counterparts do not. This study outlines results of the implementation evaluation of a program serving EPS at community colleges. These results highlight the need to conduct research early in program planning to understand the needs of the population, use these data to inform programming, be flexible in implementation, and work with students to offer activities and services that fit within their schedules.

## Objectives

Nationally, the percentage of college students with children continues to grow; currently, this population accounts for an average of 30% of the student body for community college (Noll et al. 2017). Of these nearly 3.8 million student parents, approximately 70% are mothers, and 62% are single mothers (Reichlin Cruse et al. 2019). Student parents face numerous barriers related to the challenge of parenting while attending college (Gault et al. 2016; Schumacher 2013) and expectant and parenting students (EPS) are more likely to drop out compared to their non-parenting counterparts (Horton 2015). Additionally, undergraduate EPS experience higher unmet financial need while taking classes and incur more debt one year after the completion of their education compared to non-parenting students (Gault et al. 2014). One study found insufficient financial aid, scheduling conflicts, and transportation problems are all barriers to success for EPS at community colleges (Brown and Nichols 2012). These barriers are compounded by the decreasing availability of child care, particularly at community colleges. In the decade between 2002 and 2012, the percentage of community colleges that provided on-campus child care fell from 52.4 to 46.6% (Hess et al. 2014). However, although these barriers to education completion are well documented, less information is available on how to design and implement effective programming to support this population, particularly in community college settings.

In order to design this programming we also need more information about EPS at community colleges because they have a wide variety of backgrounds and socioeconomic characteristics, making them difficult to study as a group. Despite this heterogeneity (Hawley and Harris 2005), we do know that EPS share some common characteristics. Women, particularly black women, are more likely to be student parents than men; student parents have significant unmet financial need; and nearly half of student parents work more than 30 h/week (Crispin and Nikolaou 2019; Noll et al. 2017; Reichlin Cruse et al. 2018). However, much of the data we have about EPS at community colleges is collected through national surveys: the majority of institutions do not collect these data at a college level, which inhibits researchers’ and practitioners’ ability to understand how best to serve their own populations. Although we know that EPS have many risk factors related to college success (Horton 2015; Shenoy et al. 2016; Van Rhijn and Lero 2014), we know less about what community colleges can do to alleviate some of these risks. Our paper presents results from an evaluation of a program at community colleges to address some of these barriers experienced by EPS.

### PAF Program and Implementing Sites

The federal Pregnancy Assistance Fund (PAF) is designed to assist expectant and parenting teens, young adults, and their families by providing health, educational, social, and economic services (Person et al. 2016). In 2017, the Iowa Department of Public Health was granted funding for a one-year project period through the PAF program to work specifically with EPS in the community college setting.^1^ To select colleges for participation, state public health officials met with the Executive Director of the Association of Community College Trustees, who recommended five colleges (out of 15 community colleges in the state) that had the capacity to implement a program to assist EPS. Three of these colleges expressed interest and were included in the PAF program. Demographic information about the student body for each participating college is provided in Table 1. Overall, the racial/ethnic and gender makeup of the schools is similar, but their total enrollments are different, ranging from 6,425 to 36,033. The PAF program had two main goals: (1) to increase the number of services offered to this population and (2) to increase EPS awareness of available services. The community colleges offered and promoted activities and services in five domains: (1) personal health; (2) child health; (3) self-sufficiency, education/academic, and employment support; (4) concrete support; and (5) parenting support (Iowa Department of Public Health 2017). Domains 1 and 2 encompass supports and access to health services for EPS and their child(ren). Education and/or employment supports that lead to further self-sufficiency of EPS are covered under domain 3. The concrete supports of domain 4 refer to providing tangible supports (financial and material) that allow EPS to cover basic necessities such as clothing and food and to providing assistance with government programs. Domain 5 covers supports that assist EPS with parenting skills, including communication and stress management. The state department of public health surveyed the three participating colleges prior to the start of the program and found that although colleges had preexisting programs and systems within their institutions to provide academic, employment, and parenting support, they lacked internal programs for personal health, child health, and concrete support services.

**Table 1:** Demographic characteristics of entire student body of participating community colleges, 2016 data^a^

	College A %	College B %	College C %
Race/ethnicity			
American Indian	0.4	0.4	0.3
Asian	4.1	4.9	1.2
Black	6.0	10.7	6.9
Hispanic	6.9	4.7	6.4
Hawaiian/Pacific Islander	0.1	0.1	0.1
White	75.6	71.4	76.6
Two or more races	2.3	2.5	1.6
Non-respondents	4.7	5.3	6.3
Age group			
17 and under	36.8	19.5	27.0
18–22	38.5	48.3	40.6
23–26	8.7	11.3	9.9
27–30	4.6	5.8	6.4
31–39	6.3	8.7	8.7
40–55	4.4	5.6	6.4
Over 55	0.8	0.8	1.0
Gender			
Male	47.7	48.7	44.8
Female	52.3	51.3	55.2
Total enrollment	36,033	19,902	6,425

Data from State Department of Education

Each college could tailor the activities and services offered in the program to the student population. Although each college’s program differed, sites generally followed the same format: providing concrete support, either in the form of targeted financial assistance (stipends, gas cards, or meal vouchers) or access to free food, diapers, and personal hygiene supplies, and creating or promoting activities including workshops and educational support. These workshops covered topics including social media and internet safety, domestic and intimate partner violence prevention, parenting support, job preparedness, and childhood literacy. One college chose to implement a more formal mentoring system and required consistent meetings between mentors and students to help them navigate their academic and personal responsibilities. This paper presents the methods and results of our implementation evaluation of the PAF program in community colleges in a rural Midwestern state implemented during the 2017–2018 academic year. We focused on implementation evaluation because of the shortened project timeline and because we did not believe implementation would be long enough to measure any tangible outcomes. Instead, we wanted to understand the process and the factors that contribute to program success. In order to provide the most helpful information to the colleges, our evaluation focused on collecting data that could be used to improve future efforts (Durlak and DuPre 2008). Therefore, we designed our evaluation to capture the programmatic decision-making process and the perspectives of multiple stakeholders on the program. Our data collection plan addressed the following research questions:

RQ 1. Was the program implemented as intended?

RQ 2. What were the barriers to program implementation and participation?

RQ 3. What changes were made during implementation as a result of identified barriers, if any?

RQ 4. What were the facilitators of program implementation?

Answering these research questions provides much needed insight into working with EPS, and our results could be helpful for future implementers and evaluators working with this population.

## Methods

The evaluation team designed a multicomponent implementation evaluation to assess the PAF program planning process and the barriers and facilitators to implementation. Team members collected data from a variety of sources over the program year, with the goal of triangulating data sources to fully understand implementation. The University of Iowa Institutional Review Board determined that all data collection activities related to the PAF program were not human subjects research. This determination was made because all data collection was being conducted for evaluation and program improvement purposes. We included language in recruitment materials for each data collection that explained to participants (EPS and program administrators at community colleges) the voluntary nature of their participation and that information would be reported in aggregate and kept confidential. Evaluation activities consisted of developing and implementing tracking tools at each college (RQ 1) and collecting and analyzing the results of student participant surveys (RQ 1), student interviews (RQs 1–3), and program administrator interviews (RQs 2–4). We did not perform any analysis comparing results across colleges because the program was implemented differently in each college, and our research questions focused on broad facilitators and barriers to implementation rather than specifics of the program.

### Tracking Tool

The evaluation team developed a tracking tool for community college staff to record EPS’ participation in and receipt of PAF services and activities during the grant period. Using a Microsoft Excel spreadsheet, staff recorded demographic information about participants (age, gender, number of dependents, and nationality) and their participation in activities or receipt of services. Staff also recorded aggregate data on the number of students who attended on-campus events in situations in which they could not collect individual-level data. The evaluation team was available throughout the program period to provide technical assistance on using the tracking tool. Community college staff sent tracking data to the evaluation team monthly. At the end of the program period, the evaluation team aggregated tracking data from all three colleges to determine total counts of EPS receiving services/activities. The data contained records for 875 unique individuals, and we report both the number of unique individuals who received services and the total number of times a service was accessed.

### Student Participant Survey

The evaluation team developed a 10-minute online survey to measure EPS’ satisfaction with and to elicit feedback on the PAF program. The survey asked about which types of services they received through PAF, how satisfied they were with those services, suggestions for potential changes to the program, and demographic information about themselves and their families. At the completion of the program period, community college staff distributed the survey link to students with a record of PAF participation from the tracking tool. Data collection occurred over the course of three weeks, and students who completed the survey were mailed a $10 gift card. A total of 202 EPS completed this survey, and responses were distributed among the three colleges (college A contributed 22.8% of responses, college B had 32.2%, and college C had 45.0%). We can estimate that 23.1% of participants who received services captured by the colleges’ tracking sheets responded to this survey, however we are unable to calculate a definitive response rate because multiple people at each college were involved in the distribution process. Moreover, we are unsure if every student with a record of participation received a survey or if the survey was forwarded to others. We downloaded data from Qualtrics and used SPSS v. 25 to calculate frequencies and percentages for all variables.

### Student Interviews

To complement the student participant surveys, the evaluation team conducted interviews with program participants. The team designed an interview guide to further understand the EPS’ participation, services received, satisfaction with services, and barriers to participation (Appendix 1). Community college staff were asked to identify and provide contact information for potential interview participants that they knew had interaction with the program over the course of the year. In order to be flexible with recruitment, the inclusion criteria for the interview were that students had received at least one service through the PAF program or participated in a PAF activity; however, we asked staff to identify the students who they knew had significant interaction. EPS received a $25 gift card for participating in an interview. Trained interviewers conducted interviews via telephone and recorded them for transcription by a third-party service. Across the colleges, staff provided 33 names of EPS; of these individuals, 14 EPS completed an interview, 13 females and 1 male. Five interviews were completed with students from College A, four with students from College B, and four with students from College C. Interviews ranged from 6 to 25 min, averaging 15 min. Thematic analysis (Braun and Clarke 2006) was used to identify overarching themes from the interviews. Initial codes and a codebook were generated by two team members, and two other team members coded the transcripts. Coded transcripts were then analyzed to determine overarching themes, which are reported here.

## Program Administrator Interviews

The evaluation team interviewed the key program administrators responsible for implementing the PAF program at all three community colleges (Appendix 2). Nine interviews (three program administrators from each college) were completed. Administrators were asked to reflect on successes and challenges of both program implementation and the program planning process. Interviews averaged 38 min and ranged from 13 to 43 min. Interviews focused on the process of planning and implementing activities and services and the programs’ challenges and successes. Interviewers conducted the interviews via telephone so they could be recorded and transcribed, and transcripts were coded thematically by the evaluation team using the same method used for the student interviews.

Following all interview quotes, we used notation to differentiate between quotes from each of the community colleges, the students, and the program administrators.

## Results

We present the results below in order of our four implementation evaluation research questions.

RQ 1: Was the program implemented as intended?

Each college was expected to provide services in all five service domains and in total, community colleges initially projected 350 EPS would be served in the first year of the program. To assess implementation, we used the colleges’ tracking data and results from the student participant survey. Table 2 presents the demographics of EPS who received services during the program period and those who participated in the survey. To best understand the extent to which the program was implemented as intended, we can look at the tracking tool’s record of what services and activities were provided and utilized. Across the three colleges, 875 unique individuals received at least one type of service or participated in one activity-, far exceeding the colleges’ initial projections. Table 3 presents services/activities provided by the colleges and how many times each was received or the number of times students participated in activities. Although all colleges provided various activities/services in each of the five domains, students most utilized services that fall in the concrete support domain.

**Table 2:** Demographic information for respondents in PAF program evaluation activities by data collection activity

	Tracking tool (n = 875)		Student participant survey (n = 202)	
	n	%	n	%
Gender				
Female	553	63.2	167	84.3
Male	182	20.8	29	14.6
Other	N/A	N/A	0	0.0
Prefer not to answer	N/A	N/A	2	1.0
Not reported	140	16.0	N/A	N/A
Age				
18–24	N/A	N/A	34	16.8
25–34	N/A	N/A	92	45.5
35–44	N/A	N/A	68	33.7
45–54	N/A	N/A	4	2.0
55–64	N/A	N/A	0	0.0
15–19^a^	37	4.2	N/A	N/A
20–24	68	7.8	N/A	N/A
25 +	548	62.6	N/A	N/A
Not reported	222	25.4	N/A	N/A
Race/ethnicity^b^				
American Indian/Alaska Native	19	2.2	5	2.5
Asian	0	0.0	3	1.5
Black/African American	306	35.0	61	29.9
Hispanic/Latino	86	9.8	15	7.8
Middle Eastern/North African	0	0.0	1	0.5
Native Hawaiian/Pacific Islander	0	0.0	0	0.0
White/Caucasian	278	31.8	113	55.4
Other	N/A	N/A	3	1.5
Prefer not to answer	N/A	N/A	8	3.9
Not reported	194	22.2	N/A	N/A
Foreign-born	289	33.0	75	37.1
Expectant and parenting				
Have one or more child(ren)	852	97.4	192	97.0
Expectant at time of survey^c^	90	10.3	37	18.8

^a^The community colleges recorded different age categories on their tracking sheets; in many cases, age data were missing for participants

^b^Respondents were instructed to check all that apply

^c^Expectant refers to the respondent or the partner of the respondent

^d^Information for the tracking tool and student participant survey were collected at different times and for different purposes, therefore there is not overlap in all demographic categories, any instance where a question was not asked in a data collection is denoted with a N/A (not applicable)

**Table 3:** Selected tracked services and activities received by PAF participants (n = 875)

	Unique individuals who received service	Total number of times service was accessed or total dollar amount provided
Personal health		
Breastfeeding supplies	16	48
Visits to dental clinic	30	30
Times lactation rooms were utilized	Unknown^a^	625
Child health		
Children’s visits to dental clinic	Unknown^a^	42
Children participating in recreation events	101	982
Education and employment		
Textbooks purchased for individuals	63	300
Times individuals received course supplies	86	106
Times individuals received reimbursement for graduation cap and gown	16	16
Times individuals participated in college or career planning	21	24
Students who met with campus advisor	174	498
Hours of academic tutoring provided for high school equivalency test	Unknown^a^	378
Concrete support		
Gift cards distributed and intended for grocery shopping	84	98
Dining/meal vouchers individuals received for their child	34	834
Visits to food pantry	97	350
Gas cards received distributed to pay for school-related transportation	172	$13,443
Diapers or other child care items received from food pantry	139	420
Personal care and hygiene items disbursed from food pantry	87	287
Parenting support		
Times individuals attended educational seminar	202	N/A^b^
Times individuals received parenting books	100	198
Number of individuals who participated in violence prevention seminars	98	N/A^b^

^a^Unknown indicates that colleges were unable to track individual utilization of a particular activity or service

^b^Not applicable because an activity only occurred once

**Fig. 1:**
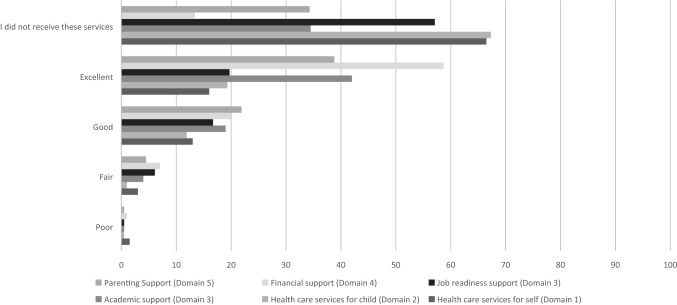
Percentage of respondents reporting satisfaction and non-utilization of services/activities provided by PAF program

While colleges were able to provide services in all domains, responses from the student participant survey indicated that not all students received services or participated in activities in each of the domains. Overall, EPS reported receipt of or participation in some of the services/activities in some domains more than others (Fig. 1). For example, nearly two-thirds of respondents reported not receiving any health care services for themselves or their children (domains 1 and 2), whereas only 13.4% reported not receiving any financial support (domain 5). However, students who received support consistently reported high satisfaction with services/activities in all domains.

RQ 2: What were the barriers to program implementation and participation?

Program administrators and PAF participants reported various barriers to program implementation and participation. A primary barrier reported by both EPS and program administrators was promotion of the program. EPS reported hearing about PAF from various sources across their campus. Half of the EPS interviewed reported they heard about the program from their advisor or another staff member at their college (n = 7), and a few had learned of it either from a classmate who was receiving services (n = 2) or from an email or other online information (n = 3). Additionally, even when EPS knew about some aspects of the program—primarily the availability of financial support—they reported other aspects were not well advertised. Several of those EPS interviewed reported they had not realized the extent of activities/services offered until the end of the program. One respondent noted not “know[ing] anything about any of the services” [CCA_ST_4] beyond the financial assistance they received, and another believed the college needed to “create more awareness to…the parents who are students.” [CCC_ST_3].

Similarly, although the program reached more students than projected, administrators at all three colleges reported challenges in identifying and recruiting eligible EPS. The only mechanism for identifying EPS was if students had filed dependents on their Free Application for Federal Student Aid (FAFSA) record. However, pregnancy status is not included on FAFSA, and colleges knew these records were incomplete; to identify EPS, staff often had to specifically ask students. Administrators noted the “awkwardness” of asking students if they were expectant or parenting. Questions about eligibility for programming involving financial and other concrete assistance (gift cards, food pantry visits, and financial stipends) also led staff to have uncomfortable conversations about finances.

Additionally, EPS who were interviewed reported several barriers to being able to participate in PAF activities or access services provided by the program. These included class schedules and the demands of their personal lives. Four of the EPS interviewed reported that although they knew about many of the services offered on campus, they were unable to attend them because of conflicts with their class or work schedules. For EPS who took only online classes, the services available on campus were not useful. Overall, this population is very heterogeneous (see Table 2) and possesses unique needs and academic and family situations, making it difficult for the colleges to design programming that would be readily available to all EPS.

Finally, the one-year project period for the PAF grant award was a significant barrier to implementation. Previous PAF cohorts had grant award periods that allowed for a three-year implementation period; however, this award was available for one year, which greatly impacted program implementation. Colleges had originally planned to spend the first grant year assisting with data collection for needs assessment, building relationships with community organizations, and beginning to implement programming and policy changes to support EPS. However, this shortened award curtailed their ability to have a planning year and became a large barrier to implementing what they had originally planned.

RQ 3: What changes were made during implementation as a result of identified barriers, if any?

Although most of the programming was implemented as intended, staff members made several purposeful changes during the implementation period to respond to barriers encountered. These changes focused on adapting to meet EPS’ needs and responding to the significantly shortened time period. To develop programming, community college program administrators reported they implemented services/activities on a trial basis to learn what would be popular with students. For example, one administrator reported, “We did try a couple of activities that students weren’t interested in. They didn’t register, they didn’t participate, so we just kinda regrouped and then went on.” [CCC_PA_1] Administrators reported that changes were primarily made to adapt to the needs of students and externally imposed constraints that included the shortened project period.

To respond to the fact that colleges needed to condense what was originally planned for three years into one, they reported that they had to focus on “what is practical [to accomplish],” [CCB_PA_3] according to one administrator. One of the more practical options they identified was disbursing financial support to match EPS’ needs; however, financial supports that colleges could provide a student were limited. Because of restrictions on what money could be used for (i.e., gas cards and grocery store gift cards could be furnished, but students’ electricity bills and rent could not be covered by grant money), some administrators noted that some students’ needs were not able to be met. Several administrators noted having the flexibility to better match monetary support and comprehensively address all of EPS’ needs would have been helpful.

RQ 4: What were the facilitators of program implementation for community colleges?

Program administrators from all three colleges reported that having a “program champion” within their college helped to facilitate the success of program implementation. These individuals, which included faculty and staff, helped to identify and recruit participants and organize and promote activities. Administrators from each college reported the program champion’s recruitment via word of mouth and his or her promotion of activities/services were most successful in reaching potential PAF participants. Beyond these program champions, colleges relied on various recruitment methods, including word of mouth, emails, posters, social media, and print media, to advertise the program. Although administrators discussed these varied forms of recruitment for the program, in interviews, students most commonly reported that they learned about the program from community college faculty or staff.

## Conclusions for Practice

### Implications for Program Implementers

Our evaluation of PAF programs implemented at three community colleges revealed findings that may be beneficial for those who work with EPS populations in this setting. First, all colleges experienced challenges identifying and recruiting EPS. No database of EPS exists, and although it may be possible for administrators to glean some information about this group from FAFSA records, those forms have limitations and provide no tracking mechanism for EPS. We recommend that colleges collect information specifically on their expectant and parenting student population to better be able to serve them and match them with available services. The colleges in this study relied on one-on-one outreach conducted by staff champions to identify and track EPS, but because such outreach can be time consuming, having processes in place to accomplish this task would be beneficial.

The problem of identifying and reaching this population is compounded by its heterogeneity. Compared to the state’s population, demographics from all data collections revealed the sample that we have data for is ethnically and racially diverse and often under-resourced. All these characteristics make them a vulnerable population (Shivayogi 2013), and researchers, evaluators, and programmatic staff need to be aware of this status and take particular care when working with this population. Given that EPS are a vulnerable and hard to identify population, program implementers could utilize traditional sampling techniques for these types of populations, like snowball or respondent-driven sampling (Ellard-Gray et al. 2015)—but instead of recruiting individuals for research activities, the goal would be recruitment into programs.

The second finding is that program administrators need to be flexible throughout implementation, particularly in terms of choosing how to structure their program and which services/activities to implement in their colleges. This flexibility is especially important in ensuring the program is adaptable and able to be utilized by all EPS. We heard from both staff and students that time is a major barrier to program implementation. Prior research identified that female community college students in particular face multiple demands on their time, especially when they are mothers (Johnson et al. 2010). EPS should be consulted prior to activities and services planning to ensure timing works for them; conducting activities in the early morning or late evening hours could better accommodate their demanding schedules as parents and students.

Finally, although we were unable to collect data at multiple points throughout the program because of the shortened project period, we suggest that future implementation efforts incorporate more frequent data collection. All colleges reported that they were challenged to quickly adapt to the students’ needs and response to activities to best tailor the program to their expectant and parenting student population. By collecting data on student satisfaction more frequently, administrators could better respond to students’ needs and design effective and tailored programming.

### Strengths and Limitations

As evaluators, we learned enlisting community college staff as facilitators and data collectors was vital to the success of the evaluation. They were crucial in collecting all student-level data. For the student participant survey and student interviews, we relied on staff to disseminate information because we believed students would be more likely to respond to such requests if they came from their college and a name they recognized.

The ability to triangulate data from multiple sources was another strength of this study; doing so helped us understand the program from all perspectives and increased our confidence in the evaluation findings. At the end of the program year, we interviewed college staff who worked on the program at multiple levels and students who received various services. We were able to combine data from the tracking tools and our student participant survey with interview data to better identify factors that facilitated and hindered implementation. Not only could we look for consistencies and inconsistencies to cross-validate the sources, giving us confidence in our data, but also the combined qualitative and quantitative data provided a richer picture of the program.

Finally, we would like to note several important limitations to the data collected in this study. We estimate our response rate for the student survey to be 23.1% and therefore our results may not be representative of all students who participated in the PAF program. We were able to complete interviews with 42% of the students who were identified by their respective colleges and these students may not be representative of a typical program participant. Tracking participation in some activities, such as large events and receipt of some services, proved to be difficult for the colleges. This difficulty was primarily due to time constraints on community college staff to record participation, technological barriers related to aggregating data across campuses, and privacy issues. Although receiving concrete items (stipend, food pantry, gas cards, meal vouchers) was easy to track, participating in workshops and using services like lactation rooms proved difficult to track, particularly at the individual level. Therefore, the participation numbers do not fully represent all activities and services at the three colleges and undercount the actual number of activities and students served.

Despite these limitations, our findings highlight important considerations for future program implementers and evaluators of programs designed to assist EPS at community colleges. This environment is an excellent setting for interventions with this vulnerable population, and further research could expand our understanding of ways to identify EPS, comprehend their needs, and be responsive to their interests so that they receive the optimal support for their educational efforts.

## Appendix 1: Student Interview Guide

Background

We are interested in learning about you and your experience with the Pregnancy Assistance Fund program.

1. First can you tell me a little about your life as a CC student?
  {pause}.
2. What classes did you take last semester? How did that go?
3. What has been challenging?
4. So do you have children or you are expecting?
  If children, how old are your children?
5. Can you talk to me a little bit about how that works when you are in school?
  **Probe:** If younger children: do you use daycare services? If older: Do you have babysitters or family members who take care of kids after school?
6. How do you think your experience is different from your classmates who are not parents?

Services Received

7. How did you first hear about the Pregnancy Assistance Fund program at your school?
8. Can you tell me about which services you and your family received through the PAF program during this year? (May need to probe for topic areas—ex—any assistance with school, assistance with food etc.)
  **Probe** for each thing they talk about:
  What did this service do for you?

Satisfaction with PAF

9. Which of these services were most helpful to you?
  **Probe:** How were these services helpful? Who were these services helpful to (you? Your children? Your family?)
10. Which of these services were least helpful to you?
  **Probe**: Why were these services unhelpful?
11. Were there any challenges in accessing the services that were offered through the PAF program?

Other Benefits/Improvements to be made

12. What could be changed about the program to make it better for you and your family?
13. Is there anything that was different about this school year because of the PAF program?
  **Probe:** meeting other students? Learning about other services your school offers?
14. If you were in charge of creating a program for expectant and parenting students at your school what would it be like?
  **Probe:** What specific services would be offered? What do you think the pregnant and parenting students at your school would most want?

## Appendix 2: Program Administrator Interview Guide

**Implementation/Challenges and Successes**

1. We know that all three colleges have implemented the PAF program differently. To start, can you tell me about the PAF program at your school? (Probe—need to know about what they did for students, staff).
2. What was your decision making process around what activities you focused on?
  Probe: Activities for EPS? Activities for college staff?
3. Were you able to use the Needs Assessment in your activity planning process? If yes—can you talk a little bit about how you used it?
4. What were the main challenges you and your staff experienced in implementing PAF program activities at your school? (Probe—Challenges around reaching students? Challenges in having staff onboard?).
5. What steps did you take to overcome these challenges? (Probe into what they specifically said).
6. How did you choose partners to refer students to? (Probe: were these existing partnerships or new partnerships?).
  Did you encounter any challenges in establishing these referral systems? (Probe: What were these challenges and what steps did you take to overcome them?)
  Were there any challenges in identifying partnerships? (Probe—what where these challenges? What steps did you take to overcome them?)
7. How did you promote the PAF program? Be as specific as possible? (Probe: what channels did you use? Which were most successful? Least successful?).
8. Is there anything you would change about how you implemented this program? What would it be? (Probe: about how you implemented things at your school? Support from IDPH?).
9. What do you consider the biggest successes from this year? Please be as specific as you can?
  Probe: Why do you think these were successful?

**Overall**

10. If time and money weren’t an issue, what is one thing would you change about the program?
11. If another community college were to receive this grant money in the future, what advice would you give them?
12. Finally, can you talk to me about your sustainability plan for the PAF program? (Probe- ask about plans for specific activities).

## Footnotes

1 Although the 2017 PAF Funding Opportunity Announcement was released for a three-year project period, the award for PAF grantees was made for only one year.
